# Integration of Fluorescence Spectroscopy into a Photobioreactor for the Monitoring of Cyanobacteria

**DOI:** 10.3390/bios15030128

**Published:** 2025-02-20

**Authors:** Borja García García, María Gabriela Fernández-Manteca, Celia Gómez-Galdós, Susana Deus Álvarez, Agustín P. Monteoliva, José Miguel López-Higuera, José Francisco Algorri, Alain A. Ocampo-Sosa, Luis Rodríguez-Cobo, Adolfo Cobo

**Affiliations:** 1 Photonics Engineering Group, Universidad de Cantabria, 39005 Santander, Spain; 2 Instituto de Investigación Sanitaria Valdecilla (IDIVAL), 39011 Santander, Spain; 3 Ecohydros S.L., 39600 Maliaño, Spain; 4 CIBER-BBN, Instituto de Salud Carlos III, 28029 Madrid, Spain; 5 Servicio de Microbiología, Hospital Universitario Marqués de Valdecilla, 39008 Santander, Spain; 6 CIBERINFEC, Instituto de Salud Carlos III, 28029 Madrid, Spain

**Keywords:** phytoplankton, cyanobacteria, harmful algal blooms, fluorescence, photobioreactor, continuous monitoring

## Abstract

Phytoplankton are essential to aquatic ecosystems but can cause harmful algal blooms (HABs) that threaten water quality, aquatic life, and human health. Developing new devices based on spectroscopic techniques offers a promising alternative for rapid and accurate monitoring of aquatic environments. However, phytoplankton undergo various physiological changes throughout their life cycle, leading to alterations in their optical properties, such as autofluorescence. In this study, we present a modification of a low-cost photobioreactor designed to implement fluorescence spectroscopy to analyze the evolution of spectral signals during phytoplankton growth cycles. This device primarily facilitates the characterization of changes in autofluorescence, providing valuable information for the development of future spectroscopic techniques for detecting and monitoring phytoplankton. Additionally, real-time testing was performed on cyanobacterial cultures, where changes in autofluorescence were observed under different conditions. This work demonstrates a cost-effective implementation of spectroscopic techniques within a photobioreactor, offering a preliminary analysis for the future development of functional field devices for monitoring aquatic ecosystems.

## 1. Introduction

Phytoplankton are photoautotrophic organisms that perform photosynthesis using sunlight as their primary energy source, allowing them to produce their own energy reserves. For this reason, they proliferate in the surface regions of water bodies, where they have access to sunlight and the necessary nutrients. Moreover, they are indispensable for maintaining the health and equilibrium of aquatic ecosystems, acting as primary producers in food webs and making a significant contribution to oxygen production. Phytoplankton also hold considerable potential in the fields of food [1,2,3,4] and medicine [5].

However, alterations in phytoplankton dynamics can negatively impact water quality, aquatic life, and human health [6]. Activities such as agriculture, livestock farming, and urban growth contribute to the release of nutrients, such as phosphates and nitrates, into aquatic ecosystems, causing nutrient pollution known as eutrophication. Under conditions of excessive nutrient availability and favorable temperatures, cyanobacteria may undergo rapid proliferation, resulting in a phenomenon referred to as a “bloom”. When these massive proliferations reach levels that harm the environment, they are classified as harmful algal blooms (HABs) [6], and their most serious consequences include hypoxia, i.e., a deficit of oxygen in the water, and the release of dangerous toxins [7].

One of the phytoplankton microorganisms that causes the most problems in reservoirs is cyanobacteria [8,9]. Cyanobacteria produce a wide variety of compounds known as secondary metabolites, which do not serve essential functions in their own metabolism but have toxic effects on the health of exposed organisms, including humans [10]. Due to their ability to contaminate drinking water sources and water bodies used for recreational purposes, these toxins are considered one of the most significant public health risks associated with the consumption of contaminated water [11].

HABs represent a widespread and increasing phenomenon that appears to be intensifying as a consequence of climate change [12,13]. The Special Report on the Ocean and Cryosphere in a Changing Climate (SROCC) [14] from the Intergovernmental Panel on Climate Change (IPCC) highlights changes in biogeography, increased abundance, and enhanced toxicity of HABs in recent years, partly due to global warming and other human-driven factors. Overall, it is projected that the frequency of HABs, their toxicity, and the risk they pose to natural and human systems will continue to increase throughout this century.

A major challenge with HABs is their rapid onset, often with minimal warning, making it difficult to predict when a bloom may occur [15]. Consequently, the importance of their measurement and identification has increased significantly in recent years [8,16]. Continuous local-scale monitoring programs, as well as early warning systems for resource managers and stakeholders, are essential to take preventive measures and mitigate the impacts of HABs [17].

Traditional monitoring involves routine field sampling and laboratory analysis methods, including chlorophyll a measurement through pigment extraction using solvents, morphological analysis, and species enumeration [18]. These methods require a high level of expertise and are time-consuming and labor-intensive. In general, these methods tend to be costly and laborious. Currently, technologies based on optical measurements are becoming popular for this application, as they offer advantages such as being non-invasive, providing real-time results, and offering high resolution [19]. Spectral techniques allow rapid measurement of biomass or concentration, as well as phytoplankton detection and classification. Some notable studies have focused on hyperspectral remote sensing, which can be particularly useful for large bodies of water like oceans and seas [20]. However, this technology may be less accessible for monitoring reservoirs and rivers, where HABs also pose a serious concern.

Fluorescence is one of the most fundamental techniques in phytoplankton research, primarily due to the presence of pigments that confer autofluorescent properties [21]. Its non-invasive and rapid measurement capabilities make it particularly suitable for real-time monitoring of phytoplankton dynamics in both laboratory and field settings, thereby enhancing our ability to detect and respond to harmful algal blooms [22].

Given the importance of proactive, continuous monitoring of phytoplankton in aquatic ecosystems, developing new tools that enable early detection and prediction in a simple, rapid, and reliable manner is crucial. One of the main obstacles is the high variability in phytoplankton fluorescence, which is closely tied to their physiological conditions. Factors such as taxonomic composition, pigment content and ratios, nutritional conditions, growth stage, photoadaptation, and overall physiological state all modulate phytoplankton fluorescence [21]. Consequently, advancing spectroscopic techniques require a comprehensive understanding of the temporal evolution and spectral characteristics of these signals. Such knowledge is essential for enhancing instrument accuracy and ensuring effective phytoplankton detection and monitoring [23,24].

In this context, photobioreactors provide an ideal environment for investigating these processes under precisely controlled conditions [25], making them particularly useful in research related to the evolution of phytoplankton. On a small scale, they facilitate the cultivation of photoautotrophic microorganisms, allowing for the detailed study of their growth and development [26,27].

The primary objective of this study is to integrate a fluorescence module into a low-cost photobioreactor to monitor phytoplankton fluorescence under different environmental conditions. We aim to develop a simple device capable of tracking changes in spectroscopic signals during various growth states—growth, degeneration, or degradation—since understanding these effects is crucial for developing spectroscopic detection and classification tools. Additionally, this work demonstrates the versatility of optical fibers in creating fluid-based devices, laying the groundwork for evaluating the feasibility of more compact spectroscopic systems. These advances could significantly improve the detection, monitoring, and classification of phytoplankton by providing deeper insight into how culture conditions influence optical measurements.

Accordingly, Section 2 details the steps followed in conducting this work. The subsequent subsections introduce the main fluorescence characteristics of phytoplankton, describe the features of the low-cost, open-source photobioreactor employed, outline the design and fabrication of the fluorescence module implemented, and present the cyanobacteria cultures evaluated. Section 3 elaborates on the general device performance and the results of the cyanobacteria cultivation, demonstrating its potential. This developed module, which incorporates cost-effective and easily manageable electronics, serves as a promising example of an effective method for evaluating the evolution of spectroscopic signals throughout the lifecycle of phytoplankton.

## 2. Materials and Methods

This study focuses on the design and assembly of a simple fluorescence module to be implemented in the open-source photobioreactor, the Pioreactor [28]. This section outlines the fundamental framework of phytoplankton fluorescence on which the study is based, as well as the main features of the Pioreactor in which the module is implemented, including its design and assembly.

### 2.1. Phytoplankton Fluorescence

Fluorescence is the property of certain atoms and molecules to absorb light at a specific wavelength and subsequently emit it at a longer wavelength. This interaction is widely used in the study of phytoplankton as well as in HAB research [21,22,29]. Phytoplankton fluorescence originates from endogenous pigments, as these organisms contain fluorophores that confer autofluorescence [30]. Various fluorophores are relevant in the analysis of phytoplankton, and different classes possess distinct fluorophores in specific quantities. By exciting the sample at multiple wavelengths and measuring its fluorescence, phytoplankton displays different spectra based on the relative proportions of their pigments, thus enabling their classification into different spectral groups [31]. For instance, divisions such as chlorophytes are classified within the green spectral group because their fluorescence is predominantly due to chlorophylls a/b. Conversely, cyanophytes (cyanobacteria) belong to the blue spectral group due to the predominance of phycocyanins. Spectral classification of phytoplankton by fluorescence reveals that each group presents a unique spectral signature, facilitating group identification through fluorescence measurements [29].

Among the phytoplanktonic microorganisms responsible for HABs in reservoirs, cyanobacteria are especially significant [6,9,11]. Due to their importance in HAB formation and the need to continue developing technologies for their detection, cyanobacteria will be used as test organisms for the device developed in this study. For this purpose, it is essential to understand their fluorescence characteristics first.

In relation to the spectral groups mentioned above, we noted that cyanobacteria belong to the spectral group commonly referred to as blue, where phycocyanin is the most relevant fluorophore. Cyanobacteria possess supramolecular complexes on the surface of their thylakoid membranes, known as phycobilisomes, which serve as light-harvesting antennae. These complexes consist of phycobiliproteins, such as phycocyanin, assembled into an organized structure that enhances the absorption and transfer of light energy to the photosynthetic reaction centers. Phycocyanin absorbs light within the 620–630 nm range and emits fluorescence around 650 nm. This arrangement enables a directional transfer of the absorbed energy toward chlorophyll a in photosystem II, thereby maximizing photosynthetic efficiency [21]. It allows cyanobacteria to efficiently use the available light in their environment, especially in aquatic settings where certain wavelengths may be limited. Therefore, it is expected that cyanobacterial autofluorescence is primarily governed by phycocyanin and that chlorophyll a may also contribute to some extent.

**Figure 1:**
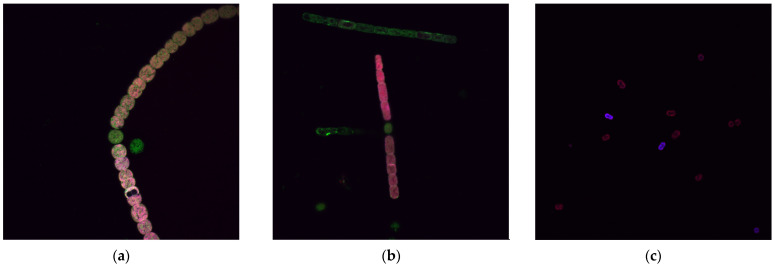
Fluorescence images of the cyanobacteria: (**a**) *Dolichospermum crassum* UAM 502; (**b**) *Aphanizomenon* sp. UAM 588; (**c**) *Microcystis aeruginosa* UAM 253. Images were acquired using a NIKON A1R confocal fluorescence microscope with a 60× objective lens. Excitation was performed with laser lines at 405 nm, 488 nm, and 561 nm, and the resulting fluorescence signals were overlaid and displayed in different colors.

**Figure 2:**
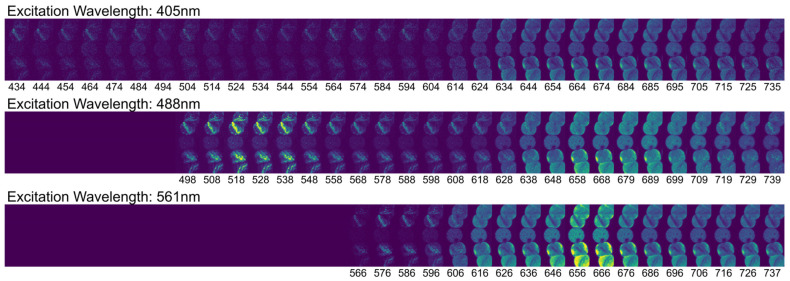
Spectral emission collection of *Dolichospermum crassum* UAM 502 cyanobacteria under 405 nm, 488 nm, and 561 nm laser excitation. The emission images were acquired at the wavelengths indicated in nanometers at the bottom of each image. All images were acquired using the NIKON A1R confocal microscope with a 60× objective.

For a rapid preliminary characterization, the autofluorescence of several available cyanobacterial species was examined using a Nikon A1R confocal fluorescence microscope. This inverted microscope was equipped with three laser lines (405 nm, 488 nm, and 561 nm) to excite the samples. Figure 1 shows fluorescence images obtained from different cyanobacterial species. Additionally, the instrument allows for spectral scanning, enabling observation of fluorescence emission when excited at the specified laser wavelengths. Figure 2 presents these spectral scans for *Dolichospermum crassum* UAM 502 cyanobacteria. By examining these images, we can identify the primary excitation wavelengths, the corresponding emission wavelengths, and the spatial distribution of fluorescent pigments within cyanobacterial cells.

**Figure 3:**
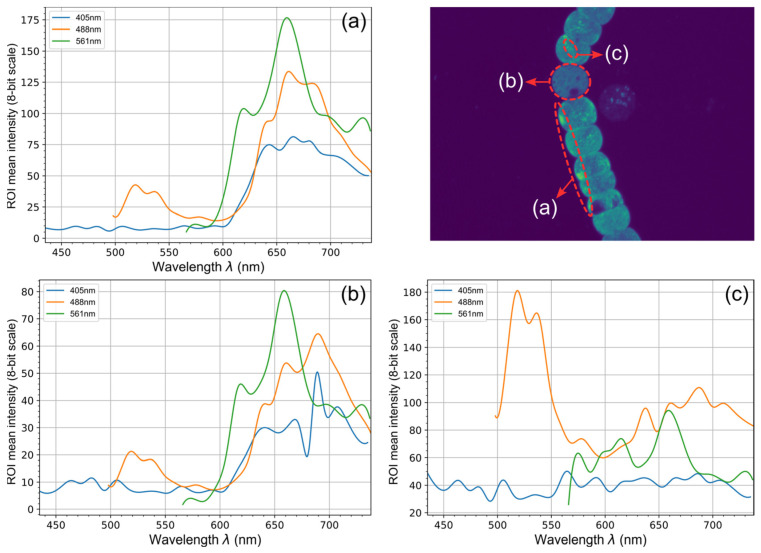
Average intensity spectra of autofluorescence from different ROIs of the cyanobacterium UAM502, obtained from the spectral scan images acquired with the NIKON A1R confocal microscope: (**a**) outer membrane of vegetative cells, (**b**) heterocyst, and (**c**) internal structures of vegetative cells.

By plotting the average intensities of some ROIs (regions of interest) from the spectral scan images, we obtained the spectra shown in Figure 3. In this figure, we can observe the different autofluorescence emissions exhibited by these organisms. Although this method does not provide high resolution, it offers a quick and effective way to visually assess the autofluorescence of cyanobacteria.

In the outer membrane of vegetative cells, a prominent emission appears at approximately 656 nm when excited with the 561 nm laser line, which can be related to the phycobilisomes present in the thylakoids. A weaker contribution, possibly attributable to chlorophyll a excitation, is also evident. Chlorophyll becomes especially noticeable in heterocysts, where under the bluer excitations at 488 nm and 405 nm, the emission peak shifts to around 689 nm. Additionally, within vegetative cells, several internal structures fluoresce at about 518 nm under 488 nm excitation; these structures are also clearly visible in the second image collection of Figure 2.

Taking this into account, we can broadly conclude that the most prominent fluorescence, and therefore the primary one we should exploit, is that related to phycobiliproteins at around 656 nm. This may be accompanied by a minor contribution from chlorophyll a at slightly longer wavelengths.

### 2.2. Pioreactor

It is necessary to create an appropriate environment by controlling the most critical cultivation conditions. These conditions can be artificially recreated in the laboratory using bioreactors to effectively study the growth and evolution of phytoplankton. Bioreactors can vary in size and configuration. Since phytoplankton organisms are photoautotrophic, photobioreactors are employed for their cultivation. These are specialized bioreactors that cultivate photosynthetic organisms such as algae and cyanobacteria using light as an energy source. These microorganisms develop in a growth medium that provides the necessary nutrients and promotes their growth and metabolism.

In this work, due to their low cost and the ease of modification for our specific purposes, an open-source photobioreactor was used: the Pioreactor. The Pioreactor is a low-cost bioreactor that is part of a project advocating for the democratization of biology [28].

The Pioreactor consists of two main components: a Raspberry Pi microcomputer (a compact, affordable, single-board computer widely used in embedded systems due to its versatility and connectivity options) and Pioreactor hardware. The hardware includes a Pioreactor’s custom HAT (Hardware Attached on Top) that connects to the Raspberry Pi, expanding its functionality for specific tasks required by the Pioreactor. The device is compact, measuring approximately 9 × 6 × 12 cm. With the vial serving as the culture vessel, the total bioreactor volume is 20 mL, and the working volume is about 15 mL.

**Figure 4:**
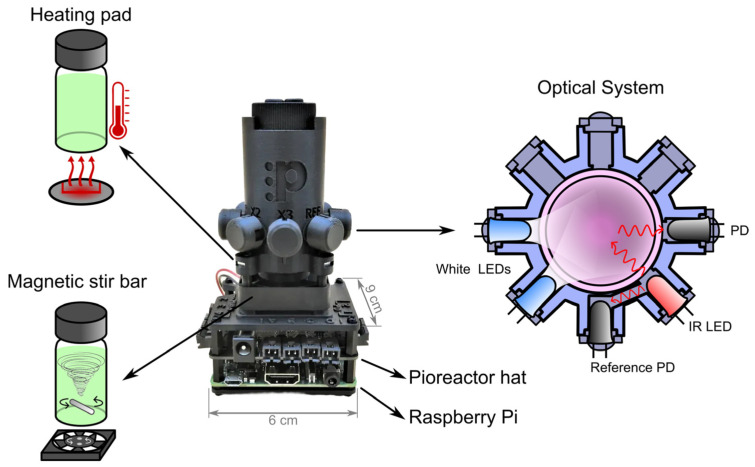
The Pioreactor is an open-source, 3D-printable photobioreactor built around a Raspberry Pi with a custom HAT. It integrates three main systems: a magnetic stirrer, a thermostat, and an optical system.

As illustrated in Figure 4, the Pioreactor features three main systems: a magnetic stirrer, a thermostat, and an optical system. The magnetic stirrer uses a 15 mm magnetic bar inside the vial. Agitation is achieved via a fan with two magnets positioned beneath the vial, enabling mixing at speeds ranging from 100 to 1000 RPM, controlled through a feedback loop. Beneath the vial, integrated heating elements and temperature sensors stabilize the culture temperature. This setup allows temperature control from ambient conditions up to approximately 20 °C above ambient, with both manual and automatic adjustment options. The optical system uses a near-infrared (IR) LED emitting at approximately 900 nm, along with two photodiodes (PD), to measure optical density via light scattering or transmission. The sensors can be adjusted for different scattering sensitivities, providing real-time monitoring of culture conditions and recording data every five seconds.

Additionally, it is possible to incorporate 5 mm white LEDs with currents up to 100 mA, thereby converting the Pioreactor into a photobioreactor for cultivating photosynthetic microorganisms. As is well known, microorganisms such as cyanobacteria require day/night cycles for their development. These LEDs enable the control of the photoperiod. All components are integrated into a web interface to control the Pioreactor. The Pioreactor measures optical density in real time. Nearly all data and system changes are recorded in an integrated SQL database and can be easily exported to CSV format.

### 2.3. Fluorescence Sensor Design

For the implementation of fluorescence in the photobioreactor, at least two essential components are required: (1) a light source to excite the sample and (2) a sensor to collect the resulting emission. In the subsequent sections, we will present the designed excitation module, the fluorescence collection methodology, and the integration of these elements into the Pioreactor system, both at the hardware and software levels.

#### 2.3.1. Excitation Module

**Figure 5:**
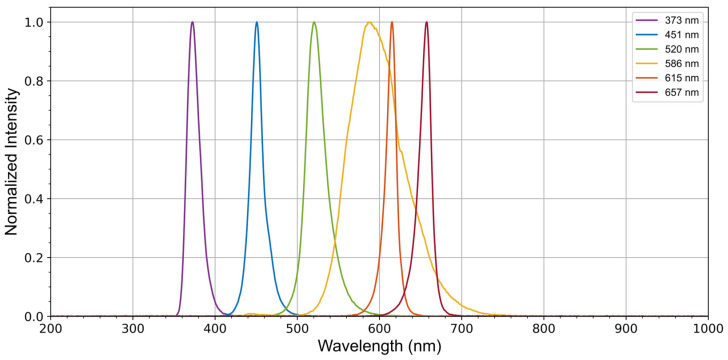
Emission spectra of the LEDs used in the excitation module. These spectra were actually measured from the light emitted directly from the plastic optical fiber connected to the LEDs.

As observed in the characterization of phytoplankton fluorescence, different spectral classes respond to distinct excitation and emission wavelengths depending on the fluorophores they contain. Although not all excitation wavelengths may be necessary for monitoring cyanobacterial fluorescence specifically, the developed module is designed to accommodate general phytoplankton by integrating six different LEDs. These LEDs cover significant wavelength ranges relevant to various spectral classes. The six commercial LEDs used are listed in Table 1, which also includes the peak wavelength of each LED, obtained by characterizing their emission spectra, as shown in Figure 5.

Due to the small size of the photobioreactor, there is insufficient space to integrate all the excitation LEDs adequately. For this reason, optical fibers are used to guide the light from each LED to the appropriate position for the correct excitation of the vial. The optical fibers employed are CK-60 Eska™ High-Performance Plastic Optical Fibers, with a diameter of 1.5 mm and a high numerical aperture of 0.5, providing greater light collection capacity and alignment tolerance.

**Figure 6:**
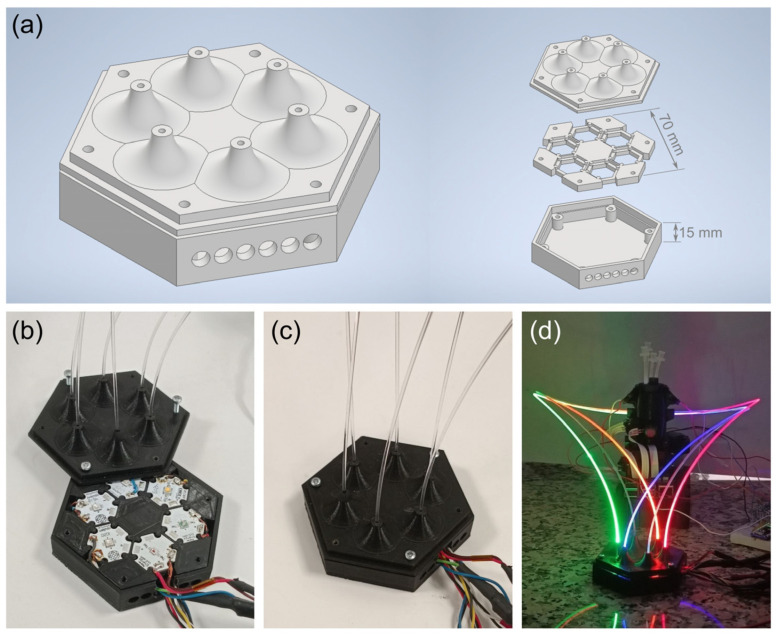
LED housing designed for fluorescence excitation. (**a**) 3D representation of the assembled housing and its three individual components. (**b**) Opened housing displaying the LEDs. (**c**) Assembled housing with the integrated optical fibers. (**d**) Excitation module with all LEDs activated, guiding light toward the vial.

For convenience and proper coupling of LED light to the fibers, the components shown in Figure 6 were designed. These components form a box that can accommodate up to six LEDs. The top cover features narrow openings through which the plastic fibers are inserted, ensuring alignment with the LEDs. The three-piece structure of the housing allows for easy replacement of the desired LEDs.

#### 2.3.2. Spectra Acquisition

For fluorescence collection, a UV/SR-VIS (200–1100 nm) optical fiber with a 600 µm core diameter from Ocean Optics was employed. This fiber guides the emitted fluorescence light to the Broadcom Qmini AFBR-S20M2WU spectrometer (Broadcom, San José, CA, USA). The spectrometer is a compact, high-resolution device. Key characteristics of the spectrometer include a spectral resolution of 1.5 nm, a detection range spanning from 225 nm to 1000 nm, and a high sensitivity suitable for low-intensity fluorescence signals. Using this system, we can obtain the fluorescence spectra of the samples, allowing for the detailed analysis of autofluorescence signals. The optical fiber and the compact design of the spectrometer allow for easy and convenient integration into the photobioreactor.

#### 2.3.3. Implementation

**Figure 7:**
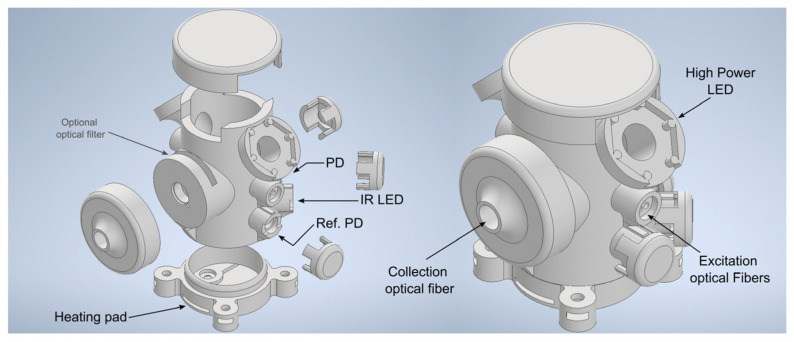
Redesign of the Pioreactor mount for implementing the fluorescence module. Three lateral holes are provided on each side for the plastic fibers to deliver the excitation light. On the same horizontal plane, oriented at a 90° angle, there is an opening for connecting the silica optical fiber leading to the spectrometer. On the upper sides, new supports are included for the white LEDs of the photoperiod.

To integrate all the systems, the photobioreactor was redesigned and 3D-printed using black PLA (polylactic acid) filament. Figure 7 shows the redesigned photobioreactor mount. The LED excitation for fluorescence is delivered via an optical fiber placed in the excitation module, guiding the light through lateral openings in the photobioreactor mount that direct it toward the side of the vial. On a horizontal plane at a 90° angle to these fibers, the silica Ocean Optics UV-VIS optical fiber is positioned to collect the fluorescence and direct the light to the AFBR-S20M2WU Broadcom Qmini spectrometer. Although we do not use it in this study due to the way we conduct and process our measurements, the mount includes a slot in front of the fiber that allows for easy insertion of different optical filters. Depending on the specific study or experiment, if the excitation signal does not need to be collected and a clearer fluorescence measurement is desired, an unmounted 25 mm diameter filter can be inserted.

Because colloids typically scatter light strongly, transmission measurements are not ideal, as scattered light can mask the true fluorescence. Therefore, this 90° detection is commonly preferred in fluorescence studies of colloids, since it reduces the impact of scattered light and enhances the selectivity of the fluorescence signal.

For optical density measurement, which was already integrated into the Pioreactor, openings for the IR LED and photodiodes have been arranged similarly to the original design. Additionally, to provide greater control and versatility over photoperiod illumination, the 5 mm white LEDs have been replaced with high-power LEDs (L2-PGC2-S de Bivar Inc., Irvine, CA, USA) mounted on the top of the reactor.

**Figure 8:**
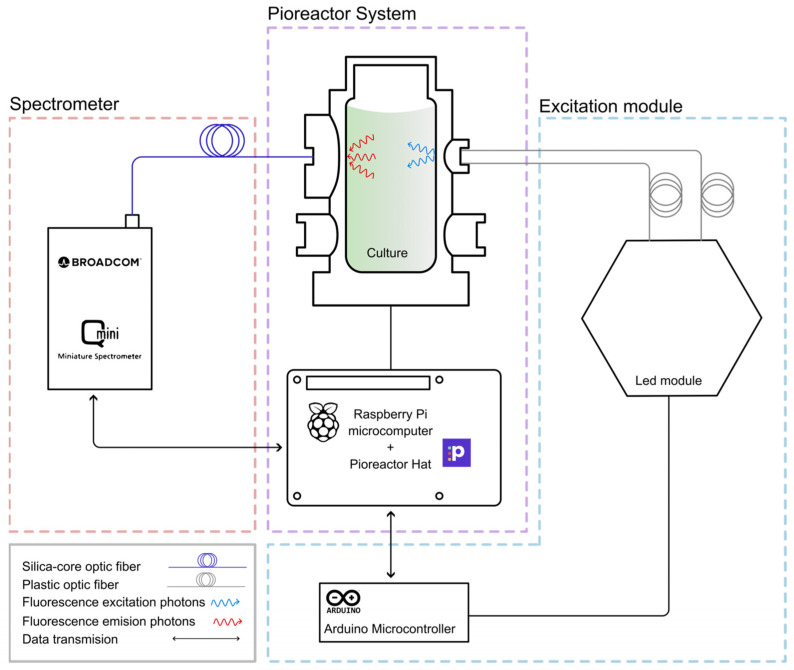
Schematic diagram of the implemented fluorescence system. The LED module is connected to an Arduino microcontroller, which communicates with the photobioreactor’s Raspberry Pi. Excitation light from the LEDs is guided to the vial through plastic optical fibers, while the resulting fluorescence is collected via a silica fiber and directed to the Broadcom mini spectrometer. The spectrometer is connected to the Raspberry Pi for spectrum acquisition and data transfer management.

Regarding system control, all operations are managed from the Raspberry Pi 4 Model B, where the Pioreactor image is installed. Figure 8 schematically illustrates the integration of the fluorescence module, which consists of two main components: (1) the excitation module and (2) the spectrometer. The LEDs in the excitation module are controlled by an Arduino Nano 33 BLE microcontroller (Turin, Italy). A simple code is used to control LEDs of different wavelengths through basic commands sent via the serial port, with the Arduino connected to the Raspberry Pi by USB. The light from each LED, corresponding to different wavelengths, is guided by a plastic optical fiber to the side of the vial, illuminating the sample. The light emitted by the sample is then collected by a silica optical fiber and delivered to the spectrometer. The spectrometer, in turn, is connected to the Raspberry Pi via another USB port. We can control it and obtain spectral data using our own Python program, which is facilitated by the software development package provided by the Broadcom spectrometer and the NioLink protocol. The high-power LEDs for the photoperiod are connected to the specific input on the Pioreactor HAT, and their control is already integrated into the Pioreactor software (v24.10.29), accessible through the default user interface.

The Python-based plugin is responsible for managing all tasks required for the proper functioning of the culture, performing fluorescence measurements during specified time periods, and controlling the selected LEDs. It has also been integrated into the Pioreactor user interface for more convenient and straightforward operation. This integration adds a new job to the interface, which, upon initiation, begins fluorescence monitoring.

Once we start the culture as usual in the Pioreactor and activate its various functions—such as optical density measurement, temperature control, stirring, and photoperiod—we can enable fluorescence monitoring. This process begins by pausing any ongoing jobs that could interfere with the measurements (e.g., optical density readings) and ensuring that the white LEDs for the photoperiod remain off. It then turns on the corresponding excitation LEDs and captures the necessary spectra.

By default, the plugin is programmed in a general manner to use all six available excitations, switching on each LED one by one while capturing the spectra with the spectrometer. Once the measurement round is complete, the plugin restores the previously paused jobs so that normal operation can continue until the next measurement cycle begins after the designated interval.

Additionally, all obtained spectra are saved in the storage folder of the Pioreactor’s memory, allowing them to be easily retrieved for more thorough analysis. At the same time, real-time charts of the measured fluorescence over time are provided in the user interface, enabling users to observe the progress as it occurs.

One of the main issues we encounter is the contribution of scattering to increases or decreases in fluorescence. As optical density increases, scattering becomes more pronounced, and consequently, the signal measured at a 90° angle with the spectrometer also increases. To help minimize this effect, we collect two spectra during each fluorescence measurement for every excitation. One spectrum is recorded at a very short exposure time (0.1 s) to observe the LED excitation peak, while the other uses an exposure time sufficient to capture the fluorescence signal accurately. In our experiments, we determined that an exposure time of 5 s was sufficient to accurately capture the fluorescence signal from our cultures while avoiding excessively long measurements. This dual-measurement approach is necessary because a single measurement cannot simultaneously capture both the fluorescence signal and the LED excitation peak. The autofluorescence emission from the colloid is considerably weaker than the LED excitation. Due to this low efficiency, obtaining a meaningful fluorescence spectrum requires increasing the exposure time. However, this inevitably leads to the saturation of the LED peak in the fluorescence spectra shown. The interest in monitoring excitation is to determine whether a modulation in the peaks could be present (Figure S1). As expected, due to scattering effects, the higher the concentration of scatterers, the greater the intensity received by the fiber. This would also affect the emitted fluorescence, and the modulation of the fluorescence peak would behave similarly. To mitigate the influence of scattering on the measured fluorescence signal, after performing both measurements and considering the spectrometer’s linearity of intensity with respect to exposure time, we normalize the fluorescence spectrum against the LED peak in each measurement.

Therefore, the Raspberry Pi, operating with the Pioreactor software and the newly developed plugin, will control the implemented modules and devices, managing them in coordination with all other features, jobs, and automation of the photobioreactor. In this way, the system continuously provides information on the evolution of fluorescence under the chosen cultivation conditions.

### 2.4. Cyanobacteria Cultures for Performance Evaluation

As previously mentioned, to demonstrate the functionality of the developed device, we employed one of the most problematic types of phytoplankton microorganisms responsible for HABs in reservoirs: cyanobacteria. Due to their greater resilience in culture [32,33], we utilized pure, axenic cultures of *Dolichospermum crassum* UAM 502. Both the strains and the sterile growth media BG-11_0_ were provided by the Department of Biology at the Autonomous University of Madrid (UAM). The initial cultures were incubated in a temperature-controlled room maintained at 25 °C under continuous illumination for 24 h, with light intensities ranging from 70 to 130 μmol photons m^−2^ s^−1^ of photosynthetically active radiation (PAR). Each flask was regularly stirred manually to facilitate gas exchange and prevent cell sedimentation.

## 3. Experiments and Results

Once the designed system has been described, this section will focus on its functionality. First, we will examine how the fluorescence module measures the fluorescence of different substances. Then, we will assess the practical utility of integrating the fluorescence module for measuring fluorescence under various culture conditions.

### 3.1. Spectral Characterization of Different Fluorescent Substances

To evaluate the fluorescence measurement capabilities of the designed device, we tested various substances containing fluorescent compounds across the visible spectrum. Table 2 presents the fluorophores tested, their excitation and emission wavelengths as reported in the literature, and the corresponding excitation LED used for fluorescence measurement.

**Figure 9:**
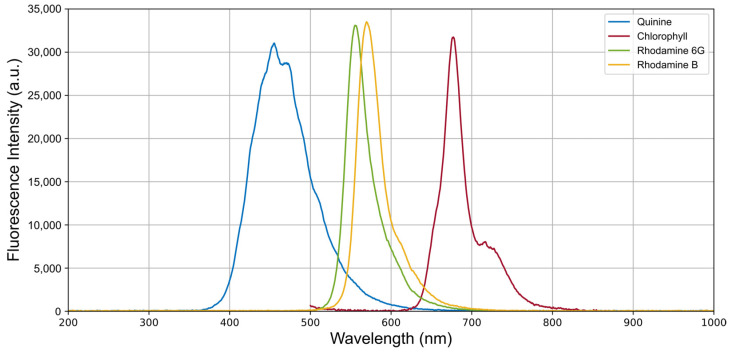
Fluorescence spectra of different fluorophores obtained using the developed device. These fluorescence signals were recorded by exciting each fluorophore with the LED whose emission peak was closest to its maximum excitation wavelength, as indicated in Table 1.

The different liquid samples were placed in the photobioreactor vial for fluorescence measurements. After activating the appropriate excitation LED, the spectrometer was used to record the fluorescence, adjusting the exposure time for each sample. The integration time was individually adjusted to obtain the best possible fluorescence spectrum capture. Figure 9 shows the fluorescence spectra obtained from the various substances, with the exposure times used being 0.37 s for quinine, 0.16 s for rhodamine 6G, 0.32 s for rhodamine B, and 2.72 s for chlorophyll.

**Figure 10:**
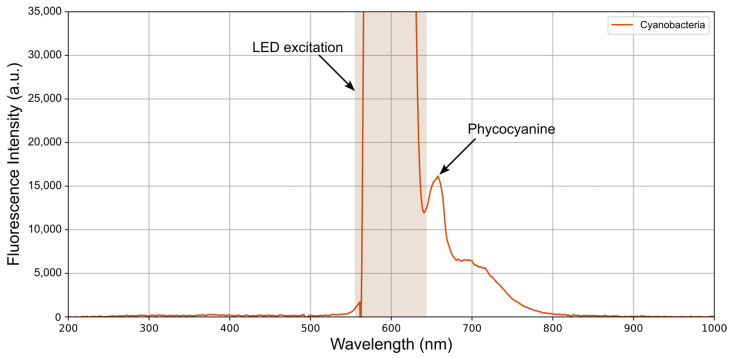
Spectrum obtained using the developed device when introducing a pure cyanobacteria culture into the vial and exciting it with a 615 nm LED. As indicated, the saturated region corresponds to the directly scattered LED light, and the adjacent peak corresponds to phycocyanin fluorescence emission by the cyanobacteria. The orange shading represents the LED excitation region.

We can observe phycocyanin fluorescence by introducing a dense, pure culture of cyanobacteria into the vial. Figure 10 shows the spectrum obtained under these conditions and captured using a 5 s integration time, where the detected fluorescence is much weaker. Furthermore, the excitation and emission peaks are very close to each other, as seen in the spectrum. The peak attributed to phycocyanin appears at around 657 nm, but there is also a slight contribution near 700 nm that could be associated with chlorophyll a, which is potentially being excited as well [39].

With this simple test, we have demonstrated that the developed device can reliably acquire fluorescence spectra from different samples. Moreover, it enables measurement of cyanobacterial autofluorescence, highlighting the phycocyanin peak at approximately 657 nm, which is consistent with the fluorescence observed under the confocal microscope described in Section 2.1.

Regarding the limit of detection (LoD), we estimated it using the sample employed in the experiments with *Dolichospermum crassum* UAM 502 grown in BG-11_0_ culture medium, based on phycocyanin fluorescence under 615 nm LED illumination. We concluded that the approximate LoD, in this case, is around 41 cells/µL (see Section S2 of the Supplementary Material).

### 3.2. Fluorescence Evolution of Cyanobacteria Culture

To ensure that microorganisms grow properly, it is essential to maintain appropriate parameters and conditions in a culture. These conditions significantly impact their development. Below are descriptions of some experiments designed to observe the evolution of the fluorescence signal of cyanobacteria under various conditions.

#### 3.2.1. Fluorescence of Cyanobacteria Under Photoinhibition Conditions

As photosynthetic organisms, cyanobacteria require adequate lighting [40]. It is important to note that they also carry out metabolic processes in the absence of light, which is vital for their growth and development. Therefore, providing an appropriate dark period is essential for their health and productivity. Additionally, the photoperiod, which refers to the duration of light and dark periods, is a crucial factor in the cultivation of cyanobacteria in a photobioreactor. To optimize their growth, a light cycle of 16 h of illumination followed by 8 h of darkness is generally recommended, although 12:12 cycles are also commonly used. These cycles promote photosynthesis during the light period and allow essential metabolic processes during the dark phase [41].

Cyanobacteria typically require light intensities ranging from 20 to 150 μmol photons m^−2^ s^−1^ within the photosynthetically active radiation (PAR) range of 400–700 nm. Early-stage or small-volume cultures can usually be maintained at lower intensities (20–50 μmol photons m^−2^ s^−1^). Cultures in active growth phases often require moderate intensities (60–120 μmol photons m^−2^ s⁻^1^). Higher intensities can be used for very dense systems, although they may induce photooxidative stress [40,42,43].

However, this intensity is highly dependent on the biovolume and other factors, such as the design or size of the photobioreactor. In our case, given the small volume of the photobioreactor, it is important to avoid excessively high intensities that can cause photoinhibition, which damages the cells.

**Figure 11:**
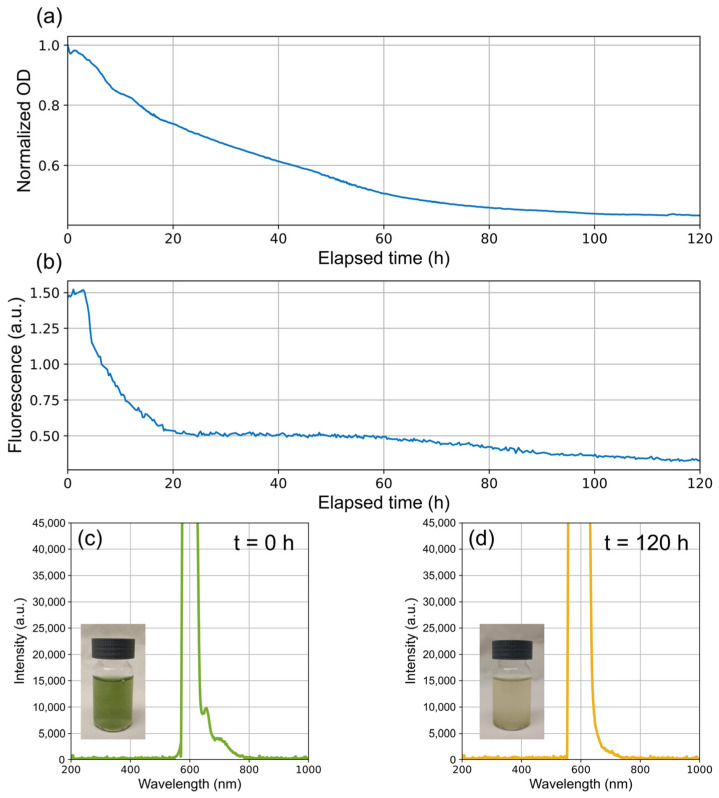
Evolution of fluorescence in a *Dolichospermum crassum* UAM 502 cyanobacterial culture under photoinhibition conditions. The figure shows (**a**) normalized optical density, (**b**) phycocyanin fluorescence, (**c**) the initial measured spectrum along with a photograph of the vial containing the culture at the beginning, and (**d**) the final measured spectrum along with a photograph of the vial containing the culture at the end of the process.

With the designed module, we conducted an experiment to observe the evolution of cyanobacterial fluorescence during a photoinhibition process caused by prolonged excessive light intensity. For this purpose, a dense culture of cyanobacteria *Dolichospermum crassum* UAM502 was introduced into the vial. The photobioreactor was initiated at an appropriate temperature of 25 °C. High-power white LEDs were turned on, providing high light intensities, which are significantly high for the small vial volume. Stirring was set at 500 rpm to properly agitate the colloidal suspension, ensuring the entire culture received prolonged illumination. Thus, optical density and fluorescence were monitored through the designed device. The results are shown in Figure 11.

In Figure 11, we can see that photoinhibition leads to a decrease in both optical density (OD) and fluorescence. With prolonged exposure, OD may drop if cells begin to die, resulting in cell fragmentation or lysis. Meanwhile, phycobilisomes are damaged by excess light, compromising the cyanobacteria’s ability to capture and transfer light energy efficiently [44]. The oxidative stress associated with photoinhibition can degrade pigments such as phycocyanin, and thus, the detected fluorescence decreases [42,43].

Initially, a slight transient increase in phycocyanin fluorescence is observed during the first few hours. This may be because, at the outset, the excess light energy is not efficiently transferred to PSII due to damage to photosynthetic proteins, causing the energy to be re-emitted as fluorescence. In the lower part of Figure 11, we see the spectra measured at the beginning and at the end of the experiment, clearly showing how the fluorescence peak is completely lost.

#### 3.2.2. Detecting Contamination in Cyanobacterial Culture

One of the most challenging aspects of maintaining microorganism cultures is contamination, as it can alter growth dynamics, compromise the viability of experiments, and lead to misinterpretation of results [45]. In the specific case of cyanobacterial cultures, early detection of undesired microorganisms is essential.

The developed fluorescence module provides information that complements optical density (OD) measurements. Thus, if fluorescence declines or disappears while OD continues to rise, it could indicate the presence of contamination in the culture. This tool allows for a quick and easy determination of whether the observed growth truly corresponds to the cyanobacteria or, instead, to contaminating microorganisms.

**Figure 12:**
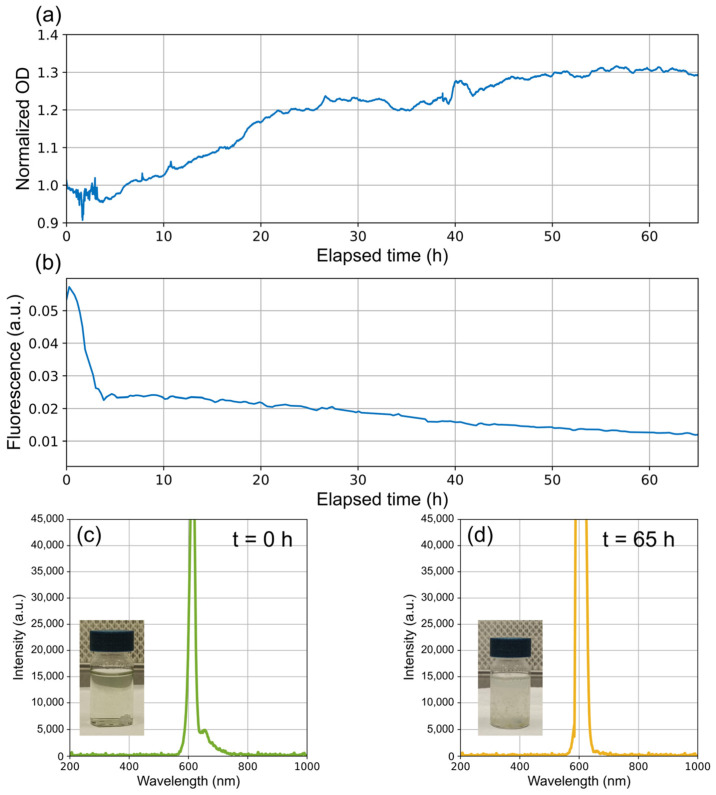
Evolution of fluorescence in a *Dolichospermum crassum* UAM 502 cyanobacterial culture during contamination. The figure shows (**a**) normalized optical density, (**b**) phycocyanin fluorescence, (**c**) the initial measured spectrum along with a photograph of the vial containing the inoculated culture at the beginning, and (**d**) the final measured spectrum along with a photograph of the vial containing the contaminated culture at the end of the process.

Figure 12 illustrates a case in which a *Dolichospermum crassum* UAM 502 culture became contaminated, possibly due to inadequate sterilization conditions. Although the culture’s optical density increased, fluorescence quickly dropped at the beginning, indicating that the rise in OD was actually caused by the growth of unwanted microorganisms. In the spectra shown at the bottom, the vial with the initially inoculated medium displays a small fluorescence peak; however, in the final spectrum, this peak has disappeared due to contamination.

## 4. Discussion

Spectroscopic techniques promise to develop cost-effective devices that effectively address harmful algal blooms (HABs). It is important to note that the autofluorescence of phytoplankton varies throughout its life cycle and under different environmental conditions. Therefore, characterizing these changes in optical properties is crucial for the proper development of spectroscopic devices aimed at the continuous detection, classification, and monitoring of these species. Implementing the simple fluorescence module developed in a photobioreactor offers a quick and practical method for studying phytoplankton fluorescence.

In this case, we focused on cyanobacteria, the main contributors to harmful algal blooms (HABs) in reservoirs. The initial fluorescence characterization of cyanobacteria was performed using a NIKON A1R confocal fluorescence microscope. Through this approach, we identified that the primary fluorophore is phycocyanin at around 656 nm, which may be accompanied by a minor contribution from chlorophyll a at slightly longer wavelengths. A more detailed analysis can also be conducted to determine the localization of these and other fluorophores. In vegetative cells, a strong emission at around 656 nm under 561 nm excitation suggests the presence of phycobilisomes in the thylakoids, with a weaker signal likely from chlorophyll a. In heterocysts, chlorophyll a becomes prominent, shifting the emission peak to around 689 nm under 488 nm and 405 nm excitations. Additionally, some internal structures in vegetative cells fluoresce at around 518 nm when excited at 488 nm.

However, although cyanobacteria are among the primary contributors to HABs, we aimed to generalize the design of the device. Phytoplankton also contains many other diverse fluorophores, which are used to classify it into various spectral groups. We tailored the excitation module to target phytoplankton in general by integrating several significant wavelengths. The resulting device consists of an excitation module with up to six LEDs spanning the spectrum to excite different fluorophores and a Broadcom Qmini spectrometer to collect the fluorescence.

To assess the device’s performance, an experiment was conducted by exciting various substances with emission peaks in different regions of the spectrum. This allowed observation of the emission spectra of quinine, rhodamine 6G, rhodamine B, and chlorophyll a, all of which showed notable fluorescence when excited with their corresponding LEDs. In the case of phycocyanin, its spectrum was detected by introducing a dense, pure culture of the cyanobacterium *Dolichospermum crassum* UAM 502 into the vial. The resulting fluorescence, obtained by exciting the colloid with the 615 nm LED, displayed a relatively weak peak located close to the excitation peak, making it more difficult to observe. This peak, at around 657 nm, can be attributed to phycobiliproteins. Moreover, a possible contribution from chlorophyll a was identified as bathochromically shifted relative to this peak.

Chlorophyll a exhibits two characteristic absorption peaks in the visible spectrum: one in the blue-violet region (around 430 nm) and another in the red region (near 662 nm). This second absorption peak interferes with phycocyanin fluorescence, which explains the appearance of fluorescence signals around 700 nm. This phenomenon is consistent with the role of phycocyanin as an antenna that enhances light capture and directs it toward chlorophyll in the form of fluorescence, which is then absorbed by chlorophyll at its second absorption maximum in the red region.

These observations demonstrate the device’s ability to record fluorescence spectra from different samples, including the fluorescence of cyanobacteria. The results for the latter are consistent with measurements obtained using the confocal fluorescence microscope, as both techniques reveal the same predominant autofluorescence.

To evaluate the overall integration and determine how the implemented module functions alongside the photobioreactor system, we cultured cyanobacteria under various conditions to observe changes in fluorescence. When exposed to excessive, prolonged illumination, we observed the effects of photoinhibition. Under these conditions, fluorescence begins to decline, and irreversible damage occurs to cyanobacterial structures, leading to cell death and loss of fluorescence. Phycobilisomes are damaged by the excess light, and the oxidative stress associated with photoinhibition can degrade pigments such as phycocyanin, resulting in a decrease in the detected fluorescence. Additionally, a reduction in optical density (OD) was noted, corresponding to cell death.

Beyond studying specific conditions, this approach can also help monitor the status and proper growth of cultures by providing information beyond what optical density measurements can offer. For instance, in some cases, cultures may become contaminated with microorganisms not originally inoculated. While these contaminants grow, the optical density may increase, yet there may be no corresponding rise in fluorescence; any initial fluorescence may even disappear over time. If fluorescence declines or disappears while OD continues to rise, this can signal the presence of contamination. Thus, this tool enables a quick and easy determination of whether the observed growth truly belongs to the cyanobacteria or, alternatively, to contaminating organisms.

The developed fluorescence module provides additional information that complements OD measurements. This capability is not limited to detecting contamination; using excitation LEDs at different wavelengths enables the analysis of samples containing multiple phytoplankton species from various spectral groups. This approach could reveal which species predominate and provide insight into their competitive dynamics, thereby supporting a more precise and efficient study of their interactions.

Regarding the challenges in developing the proposed system, the reproducibility and quality of the 3D-printed components can significantly affect the fit and optical alignment of the fluorescence module. Additionally, if a spectrometer different from the one used in our setup is employed, software adaptations, such as modifications to the existing plugin or additional packages, may be necessary. Moreover, variability in excitation, depending on the LEDs and the quality of the plastic optical fibers used, represents another factor to consider. Beyond component availability, heterogeneity in technical expertise is also a crucial aspect; the implementation and maintenance of the system require skills in electronics, programming, and optics, while successful culture growth demands at least a basic understanding of biology.

The proposed system offers a promising approach for fluorescence studies and other spectroscopic techniques while also presenting some limitations that should be taken into account. One significant limitation is its susceptibility to photobleaching, a common issue in fluorescent systems when samples are exposed to prolonged or intense excitation, making it essential to carefully adjust exposure times and intensities. Finding the optimal cultivation conditions is not a straightforward task in a device with such a small volume. In addition, depth penetration may be limited in overly dense samples, complicating the acquisition of fluorescence signals. It is also important to note that the small vial size requires working with relatively small culture volumes (about 15 mL). Finally, the nature of the sample, along with the characteristics of the medium or culture, are key factors, as significant inhomogeneities in the colloid can compromise optical measurements.

Despite these limitations, the system provides noteworthy advantages. Its capability for real-time, online measurements enables continuous monitoring of sample conditions. Moreover, the option to download raw data at any time allows for deeper analysis whenever necessary. The rapid data acquisition, achieved through a label-free approach that leverages autofluorescence, ensures that the technique remains non-destructive, preserving the evolution of the culture over time. Added to this is the system’s cost-effectiveness, along with its ease of modification, adjustment, and adaptation to specific needs, expanding its potential applications across various contexts and research fields.

Thus, this new module provides valuable information that can be used for fluorescence studies and other spectroscopic techniques. Looking ahead, future work could directly integrate other systems we are exploring to develop spectroscopic methods for cyanobacteria detection. For instance, the combination of Raman with fluorescence analysis could be very interesting, as it would overcome some limitations and offer more precise analyses, as seen in [46]. Although incorporating Raman spectroscopy would increase the system’s complexity and cost, it would constitute a promising tandem for monitoring, as well as for the detection and classification of cyanobacteria. For example, in [47], high-precision discrimination between viable and non-viable algae was achieved using Raman spectroscopy combined with multivariate analysis (PLS-DA) to assess the viability of algae present in water. Likewise, [48] demonstrates that Raman measurements are useful for detecting changes in pigment composition during cellular differentiation. To develop these types of devices, it is of great interest to incorporate the Raman technique into the photobioreactor, which would allow us to analyze how the Raman signal varies under different factors or degradation processes, similarly to what we do with fluorescence. Additionally, having an adequate characterization of the fluorescence present in the Raman spectrum is fundamental for developing precise detection and classification methods.

Recent studies, such as the one by Bi et al. [49], have proposed a method for classifying microalgae and cyanobacteria using polarized light scattering and fluorescence signals. Since the objective of our study is to observe changes in the spectroscopic signal under various imposed environmental conditions, enhancing our device to employ polarized fluorescence could be beneficial. It may help devices improve their performance by understanding how different conditions could affect the measured cyanobacteria polarized signal.

All of this can also be improved through signal enhancements, such as multiphoton techniques [50] or, in cases where identification or classification is required, by integrating machine learning algorithms [51,52].

## 5. Conclusions

The increasing development of spectral techniques for the detection and monitoring of phytoplankton requires an understanding not only of sample responses but also of their evolution over time. Harmful algal blooms (HABs) evolve and change like any biological organism, thereby altering their optical properties and various spectral responses, such as fluorescence. In this work, we propose a rapid, versatile, and low-cost methodology to conduct preliminary studies on the evolution of the fluorescence spectral responses of different planktonic organisms. We have developed a fluorescence module that integrates into a small, low-cost photobioreactor, enabling the observation of the optical fluorescence signal as the culture progresses through different conditions for phytoplankton growth. In the tests conducted with cyanobacteria, the device focuses on the evolution of the phycocyanin fluorescence peak, which emits around 656 nm. This provides additional information beyond optical density measurements. In the experiments performed, we observed a decrease in fluorescence under photoinhibition conditions and identified culture contamination by noting discrepancies between optical density and fluorescence. This device can serve as an interesting, highly promising tool to support early-stage research before the proper development of spectral devices designed for real-time HAB monitoring, which is crucial for mitigating their effects through rapid response. The optical configuration also underscores the adaptability of optical fibers in the design of fluid or liquid-based devices while also establishing a foundation for exploring the viability of this approach in smaller-scale spectroscopic systems such as lab-on-a-chip devices.

## Figures and Tables

**Table 1:** High-power LEDs used in the fluorescence excitation module, along with their radiant power and characterized peak wavelengths.

LEDs	Radiant Power	Peak Wavelength
LST1-01F06-PRD1-00 New Energy	450 mW	657 nm
XPERDO-L1-0000-00A01-SB01 New Energy	334 mW	615 nm
XPEBPA-L1-0000-00D01-SB01 New Energy	242 mW	586 nm
LST1-01G01-GRN1-00 New Energy	270 mW	520 nm
LST1-01G01-RYL1-00 New Energy	600 mW	451 nm
LST1-01G01-UV04-00 New Energy	930 mW	373 nm

**Table 2:** Excitation and emission properties of selected fluorophores tested using the developed device, along with the LED whose emission peak is closest to their maximum excitation wavelength.

Fluorophore	Max Excitation	Max Emission	Excitation LED	Ref.
Quinine	349 nm	461 nm	373 nm	[34]
Rhodamine 6G	525 nm	548 nm	520 nm	[35]
Rhodamine B	546 nm	567 nm	520 nm	[36]
Phycocyanine	620 nm	650 nm	615 nm	[37]
Chlorophyll	429 nm, 661 nm	673 nm	451 nm	[38]

## Data Availability

The Pioreactor plugin written in Python 3.11.11 and the 3D-designed parts generated during the current study are publicly available. The Pioreactor plugin can be accessed at https://github.com/borjaggarcia/fluorescence-monitoring (accessed on 16 February 2025), and the 3D designs are available on Printables at https://www.printables.com/model/1140171-led-housing-for-fiber-coupling (accessed on 16 February 2025) and https://www.printables.com/model/1151348-pioreactor-redesign-for-spectroscopy (accessed on 16 February 2025).

## Supplementary Materials

The following supporting information can be downloaded at: https://www.mdpi.com/article/10.3390/bios15030128/s1, Figure S1: LED excitation spectra acquired with a 0.1 s exposure time during the monitoring of cyanobacterial photoinhibition. The observed modulation effects are essentially due to variations in scattering. As the density of scatterers decreases, the intensity detected at 90° is lower; Figure S2. (a) Emission spectra obtained after the excitation with a LED emitting at 615 nm for different *Dolichospermum crassum* UAM 502 cyanobacteria concentrations. The emission peaks correspond to phycocyanin (657 nm). (b) Calibration curve representing the fluorescence intensity peak at 657 nm in relation to different Dolichospermum crassum UAM 502 cyanobacteria concentrations. Reference [53] is cited in the supplementary materials.
