# Supplementary material for: Integration of Fluorescence Spectroscopy into a Photobioreactor for the Monitoring of Cyanobacteria
Source: Biosensors (Basel). 2025 Feb 20;15(3):128. doi: 10.3390/bios15030128 (PMC11940672; doi:10.3390/bios15030128)
Supplement: Supplementary file 1 [file biosensors-15-00128-s001.zip › biosensors-3476435-supplementary.pdf]

Supplementary Information

Integration of Fluorescence Spectroscopy into a Photobioreactor for the Monitoring of Cyanobacteria

Borja García García[1,2], María Gabriela Fernández-Manteca[1,2], Celia Gómez-Galdós[1,2], Susana Deus Álvarez[3], Agustín P. Monteoliva[3], José Miguel López-Higuera[1,2,4], José Francisco Algorri[1,2,4], Alain A. Ocampo-Sosa[2,5,6], Luis Rodríguez-Cobo[1,2,4,*,†] and Adolfo Cobo[1,2,4,†]

1 Photonics Engineering Group, Universidad de Cantabria, 39005 Santander, Spain
2 Instituto de Investigación Sanitaria Valdecilla (IDIVAL), 39011 Santander, Spain
3 Ecohydros S.L., 39600 Maliaño, Spain
4 CIBER-BBN, Instituto de Salud Carlos III, 28029 Madrid, Spain
5 Servicio de Microbiología, Hospital Universitario Marqués de Valdecilla, Santander, 39008, Spain
6 CIBERINFEC, Instituto de Salud Carlos III, 28029 Madrid, Spain
* Correspondence: luis.rodriguez@unican.es
† These authors contributed equally to this work and share senior authorship

S1. Modulation due to scattering

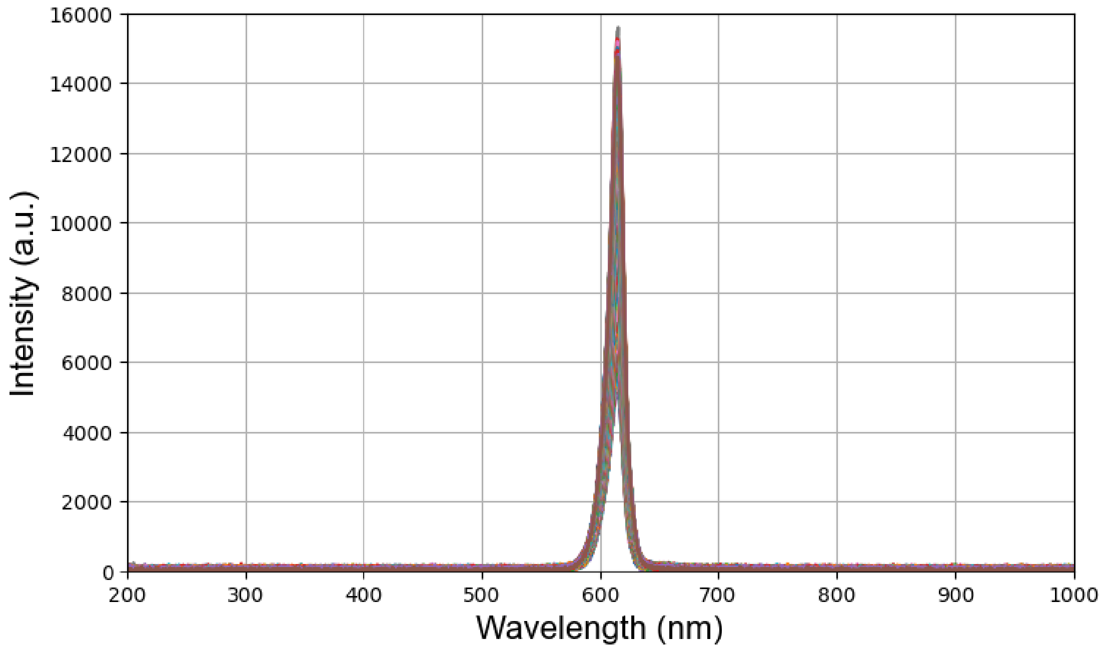

Figure S1. LED excitation spectra acquired with a 0.1 s exposure time during the monitoring of cyanobacterial photoinhibition. The observed modulation effects are essentially due to variations in scattering. As the density of scatterers decreases, the intensity detected at 90° is lower.

S2. Limit of detection

The limit of detection (LoD) will depend heavily on the type of sample, the species of phytoplankton or microorganism, the medium used, the excitation wavelength used, the efficiency of its autofluorescence, and even the specific conditions and state of the culture, as this work aims to assess.

In our case, to get an estimate, we calculated the detection limit using the sample employed in the experiments with *Dolichospermum crassum* UAM 502 in BG-11_0 culture medium, based on phycocyanin fluorescence under 615 nm LED illumination. To achieve this, serial dilutions were performed starting from a dense cyanobacterial culture, and the cell concentrations (cell/µL) were determined using a counting chamber. For filamentous cyanobacteria, such as *Dolichospermum crassum* UAM 502 in our study, counting is more complex because they form chains of varying lengths all contributing to the measured fluorescence. Due to this variability in chain lengths, counting by multicellular cyanobacteria would not be the most accurate approach. Therefore, we opted to count each individual cell within the colonies present in the counting volume.

The prepared dilutions were introduced into the vial, and fluorescence was measured with a fixed exposure time of 5 seconds, the same duration used for monitoring. The detection limit was determined by generating a calibration curve and plotting the fluorescence peak emission intensity at approximately 657 nm against the concentration of *Dolichospermum crassum* UAM 502 cyanobacteria. The values within the linear range were then used to determine the linear regression and the limit of detection using the following equation:

$$LoD = \frac{3 \times \sigma}{m}$$

Where σ represents the standard error of the response, and m denotes the slope.

In Figure S2, the emission spectra obtained for different concentrations and the calibration curve are presented. With this, we reached the conclusion that the approximate LoD, in this specific case, is around 41 cells/µL, indicating the lowest concentration that can be reliably detected above the background noise. Comparing this with other fluorescence techniques, which can reach detection limits as low as 103 cells/mL [1], our detection limit may not be the most optimal. However, it is important to consider that we are using a low-cost device, whose primary purpose is not the detection of a specific pathogen in a natural sample. Instead, it is designed for preliminary spectroscopic testing to observe how a known culture responds to imposed environmental changes.

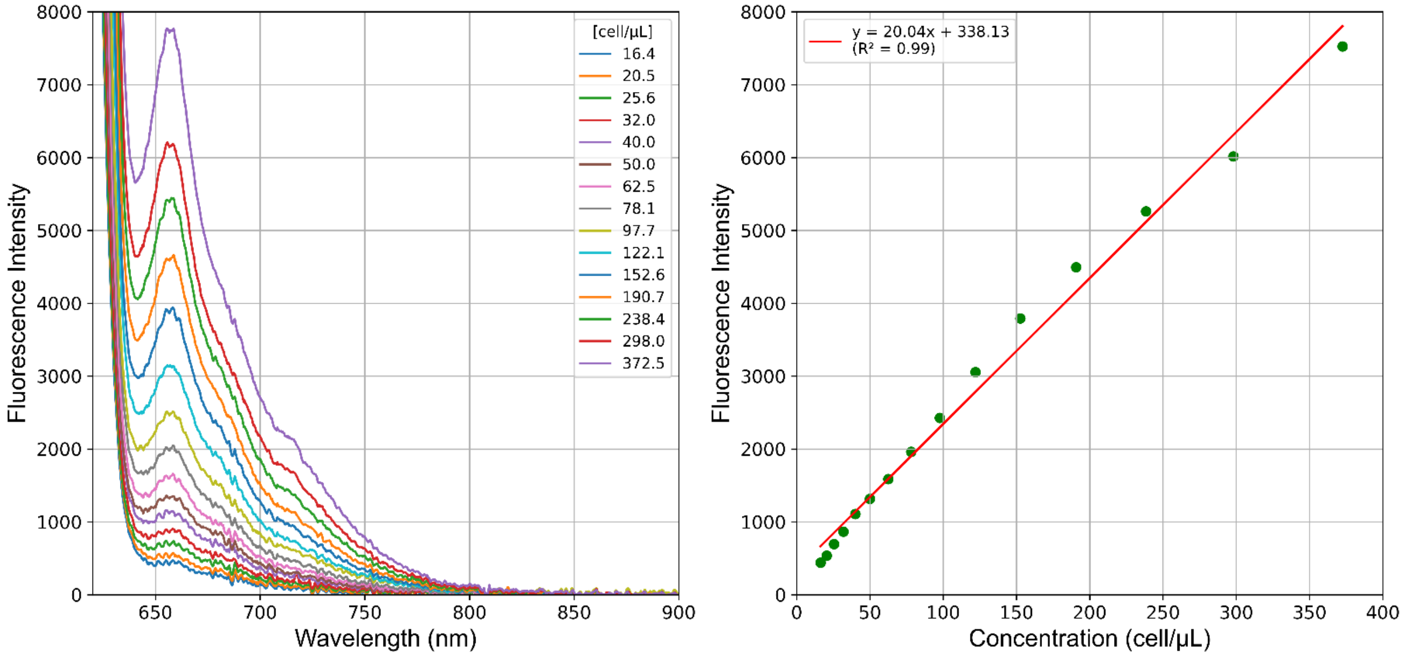

Figure S2. (a) Emission spectra obtained after the excitation with a LED emitting at 615 nm for different *Dolichospermum crassum* UAM 502 cyanobacteria concentrations. The emission peaks correspond to phycocyanin (657 nm). **(b)** Calibration curve representing the fluorescence intensity peak at 657 nm in relation to different *Dolichospermum crassum* UAM 502 cyanobacteria concentrations.

Bibliography

1. Ezenarro, Josune.J.; Ackerman, T.N.; Pelissier, P.; Combot, D.; Labbé, L.; Muñoz-Berbel, X.; Mas, J.; Del Campo, F.J.; Uria, N. Integrated Photonic System for Early Warning of Cyanobacterial Blooms in Aquaponics. *Anal. Chem.* **2021**, *93*, 722–730, doi:10.1021/acs.analchem.0c00935.
